# Supplementary material for: MetaRibo-Seq measures translation in microbiomes
Source: Nat Commun. 2020 Jun 29;11:3268. doi: 10.1038/s41467-020-17081-z (PMC7324362; doi:10.1038/s41467-020-17081-z)
Supplement: Supplementary file 10 — Supplementary Data 7 [file 41467_2020_17081_MOESM10_ESM.zip › File2/Confidence_High_Taxonomy/200068_out.krona.html]

Javascript must be enabled to view this page.

members
magnitude
magnitudeUnassigned
count
unassigned
taxon
rank

200068\_out

59

2
59
superkingdom

976
59
phylum

59
200643
class

171549
59
order

59
171550
family

genus
59
239759

species

SRS011134\_contig\_number\_37735SRS013158\_contig\_number\_343SRS014613\_contig\_number\_5643SRS014855\_contig\_number\_9742SRS014979\_contig\_number\_19803SRS015579\_contig\_number\_25484SRS015960\_contig\_number\_19288SRS017191\_contig\_number\_16244SRS018351\_contig\_number\_4130SRS018541\_contig\_number\_12132SRS020328\_contig\_number\_15168SRS020394\_contig\_number\_16203SRS021484\_contig\_number\_24718SRS021948\_contig\_number\_35837SRS022524\_contig\_number\_13437SRS043768\_contig\_number\_10089SRS045004\_contig\_number\_30685SRS045826\_contig\_number\_14615SRS049402\_contig\_number\_11988SRS049773\_contig\_number\_19573SRS050925\_contig\_number\_30856SRS051031\_contig\_number\_19306SRS053335\_contig\_number\_8229SRS055982\_contig\_number\_18027SRS064276\_contig\_number\_17062SRS075078\_contig\_number\_11824SRS075773\_contig\_number\_23926SRS075878\_contig\_number\_2516SRS076756\_contig\_number\_contig-100\_210.134234SRS077127\_contig\_number\_9930SRS077194\_contig\_number\_22503SRS077231\_contig\_number\_18674SRS077294\_contig\_number\_16231SRS098571\_contig\_number\_31595SRS098881\_contig\_number\_3638SRS104400\_contig\_number\_26856SRS105153\_contig\_number\_15595SRS140492\_contig\_number\_30305SRS142542\_contig\_number\_16913SRS142890\_contig\_number\_3594SRS144362\_contig\_number\_contig-100\_331.207335SRS144537\_contig\_number\_38333SRS146812\_contig\_number\_52710SRS147425\_contig\_number\_3144SRS148424\_contig\_number\_20411SRS149075\_contig\_number\_19081SRS893369\_contig\_number\_9735SRS893373\_contig\_number\_11895SRS893383\_contig\_number\_30308
1118061
49


SRS013800\_contig\_number\_contig-100\_781.157100SRS015960\_contig\_number\_19876SRS017916\_contig\_number\_16126SRS023583\_contig\_number\_14607SRS024435\_contig\_number\_34310SRS024625\_contig\_number\_contig-100\_5155.33111SRS056259\_contig\_number\_contig-100\_8745.127128SRS063190\_contig\_number\_8301SRS1041145\_contig\_number\_38566SRS893342\_contig\_number\_8080
species
10
1499682
